# Metabolomic Profiling of Breast Cancer Patients Undergoing Neoadjuvant Chemotherapy for Predicting Disease-Free and Overall Survival

**DOI:** 10.3390/ijms25168639

**Published:** 2024-08-08

**Authors:** Maria Cecília Ramiro Talarico, Sophie Derchain, Lucas Ferreira da Silva, Maurício L. Sforça, Silvana A. Rocco, Marcella R. Cardoso, Luís Otávio Sarian

**Affiliations:** 1 Department of Obstetrics and Gynecology, Division of Gynecologic and Breast Oncology, School of Medical Sciences, University of Campinas (UNICAMP-Universidade Estadual de Campinas), Campinas 13083-881, SP, Brazil; 2 Department of Pathology, Harvard Medical School, Boston, MA 02115, USA; 3 Brazilian Biosciences National Laboratory (LNBio), Brazilian Center for Research in Energy and Materials (CNPEM), Campinas 13083-100, SP, Brazil; 4 Division of Gynecologic Oncology-MGH Global Disaster Response, Massachusetts General Hospital, Harvard Medical School, Boston, MA 02114, USA; 5 Center for Global Health, Massachusetts General Hospital, Boston, MA 02114, USA

**Keywords:** mammary cancer, neoadjuvant chemotherapy, NMR spectroscopy, metabolome, survival analysis

## Abstract

Breast cancer (BC) remains a significant global health concern, with neoadjuvant chemotherapy (NACT) offering preoperative benefits like tumor downstaging and treatment response assessment. However, identifying factors influencing post-NACT treatment response and survival outcomes is challenging. Metabolomic approaches offer promising insights into understanding these outcomes. This study analyzed the serum of 80 BC patients before and after NACT, followed for up to five years, correlating with disease-free survival (DFS) and overall survival (OS). Using untargeted nuclear magnetic resonance (NMR) spectroscopy and a novel statistical model that avoids collinearity issues, we identified metabolic changes associated with survival outcomes. Four metabolites (histidine, lactate, serine, and taurine) were significantly associated with DFS. We developed a metabolite-related survival score (MRSS) from these metabolites, stratifying patients into low- and high-risk relapse groups, independent of classical prognostic factors. High-risk patients had a hazard ratio (HR) for DFS of 3.42 (95% CI 1.51–7.74; *p* = 0.003) after adjustment for disease stage and age. A similar trend was observed for OS (HR of 3.34, 95% CI 1.64–6.80; *p* < 0.001). Multivariate Cox proportional hazards analysis confirmed the independent prognostic value of the MRSS. Our findings suggest the potential of metabolomic data, alongside traditional markers, in guiding personalized treatment decisions and risk stratification in BC patients undergoing NACT. This study provides a methodological framework for leveraging metabolomics in survival analyses.

## 1. Introduction

Breast cancer (BC) is the most common cancer and the main cause of cancer-related deaths among women worldwide [1]. As a preoperative treatment option, neoadjuvant chemotherapy (NACT) is gaining momentum, offering benefits that include tumor downstaging, enabling less extensive surgical interventions, and reducing the distant dissemination of BC. Additionally, NACT provides oncologists with an upfront assessment of tumor chemosensitivity, aiding in treatment planning [2].

The response to NACT is believed to be influenced by various tumor and patient characteristics, with the degree of response strongly associated with survival outcomes. Notably, achieving pathological complete response (pCR) after NACT has shown a significant correlation with improved survival, particularly in patients with HER-2-positive and triple-negative BC, as well as those with luminal-type BC [3]. However, even among women who achieve pCR after NACT, distant metastatic relapse can still occur within the initial months or years of follow-up.

The ability to effectively cure BC may rely on the susceptibility of tumor cells to anticancer drugs and the development of antitumor immunity, preventing metastasis [4]. However, the factors governing antitumor immunity and the eradication of residual tumor cells following cancer treatment remain largely unidentified, leading to unexplained heterogeneity in survival outcomes after NACT. Many studies have been dedicated to identifying biomarkers associated with pCR following NACT [5,6,7,8,9,10]. In addition to NACT-associated outcomes such as pCR, a significant portion of the variability in disease-free and overall survival among NACT patients can be attributed to patient, tumor, and treatment characteristics.

In addition to this challenge, metabolomic approaches have emerged as promising avenues of exploration. Metabolomics is the scientific study of the characterization of metabolites within a given biological system, ranging from individual cells to whole organisms [11]. Valuable information about cancer biology can be uncovered, along with potential biomarkers to be therapeutically targeted [12].

In our prior research, we analyzed the metabolite profiles of pretreatment sera from women to identify potential biomarkers of the NACT response, together with immunohistochemical parameters. Our investigation focused on the serum metabolite profiles of patients with various molecular subtypes of BC who underwent NACT by employing nuclear magnetic resonance (NMR) spectroscopy. Additionally, we utilized machine learning techniques to develop classifiers that correlated the identified metabolites with BC marker expression, aiming to predict the response to NACT. Notably, these models demonstrated high predictive accuracy in anticipating the NACT response using pretreatment serum samples [13]. However, how metabolomic profiling is associated with patient survival remains underexplored. In this study, we examined the changes in serum metabolomic profiles using untargeted NMR before and after NACT in a cohort of BC patients with available follow-up data. By analyzing these profiles and the associated changes, we were able to evaluate how the serum metabolic profiles before and after NACT, as well as the changes in these profiles, are related to disease-free survival (DFS) and overall survival (OS) after BC treatment using NACT.

## 2. Results

### 2.1. Differences in the Metabolic Profiles of Patients before and after NACT

In this cohort study, 80 breast cancer patients who underwent NACT (for which the treatment details are described elsewhere [13]) were profiled for 35 metabolites before and after NACT using NMR spectroscopy. Paired fold changes for the abundances of the metabolites after NACT compared to those before NACT were calculated (Figure S1 shows the mean fold changes for each metabolite after/before NACT). Patients were followed for up to 5 years, and disease-related events (relapses, death) were recorded throughout. Using follow-up survival data, we analyzed how DFS and OS were related to metabolite fold changes. To circumvent the collinearity factor, we used the variance inflation factor (VIF)-based recursive described in the statistical section of this paper. In Table 1, we list the metabolites investigated in this study before VIF. The recursive VIF reduces the dimensionality of metabolites to those with a VIF < 3. Metabolites for which the VIF exceeded the posted threshold (VIF = 3) were recursively removed from the survival model iterations. Accordingly, Table 1 includes the final variance inflation factor (VIF) values after 13 recursion cycles (please refer to the methods section for more details). The disease-free survival coefficients were derived from the resulting and final multivariate Cox proportional hazards (CPH) model listed in Table 1 calculated using the scope of 22 remaining metabolites after VIF-based recursion.

Considering a statistical threshold of *p* = 0.10, increases in histidine (HR = 0.32; 95% CI 0.11 to 0.95; *p* = 0.04) and lactate (HR = 0.32; 95% CI 0.09 to 1.11; *p* = 0.07) levels from before to after NACT were associated with improved disease-free survival, whereas increases in serine (HR = 5.06; 95% CI 1.45 to 17.70; *p* = 0.01) and taurine (HR = 2.51; 95% CI 1.07 to 5.91; *p* = 0.03) abundances were associated with worse disease-free survival.

### 2.2. The MRSS Helps to Determine the Contributions of Metabolites and Clinical Covariates in NACT Patients

As shown in the previous analyses, four metabolites (histidine, lactate, serine, and taurine) were individually associated with disease-free survival, even after adjustment for hormones, other noncollinear metabolites, and known risk factors for breast cancer relapse (steroid receptor and HER-2 status). We thus decided to deepen the analysis and devise a metabolite-related score that could provide survival information. For that purpose, we used the survival coefficients derived for the four metabolites significantly associated with survival to derive a unique score MRSS (metabolite-related survival score), which was further used to classify patients into two groups according to their risk of relapse. Table 2 provides an overview of the key features of the 80 included patients categorized into MRSS-derived cohort subgroups of low- and high-risk patients. Most of the characteristics, namely, age, disease stage, race, age at menarche, menopausal status, hormone replacement therapy, body mass index, diabetes status, and family history of breast or ovarian cancer, were not significantly associated with the final patient status at the end of the follow-up period. However, pregnancy (previous vs. no pregnancy, *p* = 0.03) and patient final status by the end of the follow-up period (alive without disease, alive with disease, and deceased, *p* = 0.003) were significantly associated with risk subgroups. Notably, 56.5% of the patients in the high-risk group died by the end of the follow-up period, compared to only 21.1% in the low-risk group. Table 3 presents the main tumor characteristics within the cohort risk groups. None of the examined disease features, such as histological grade, Ki67 count, HER-2 status, tumor size, regional lymph node compromise, metastasis, or hormonal receptor status, were associated with MRSS risk status.

### 2.3. The MRSS Can Distinguish between Patients with Low- and High-Risk of Relapse and Even Overall Survival

**Figure 1:**
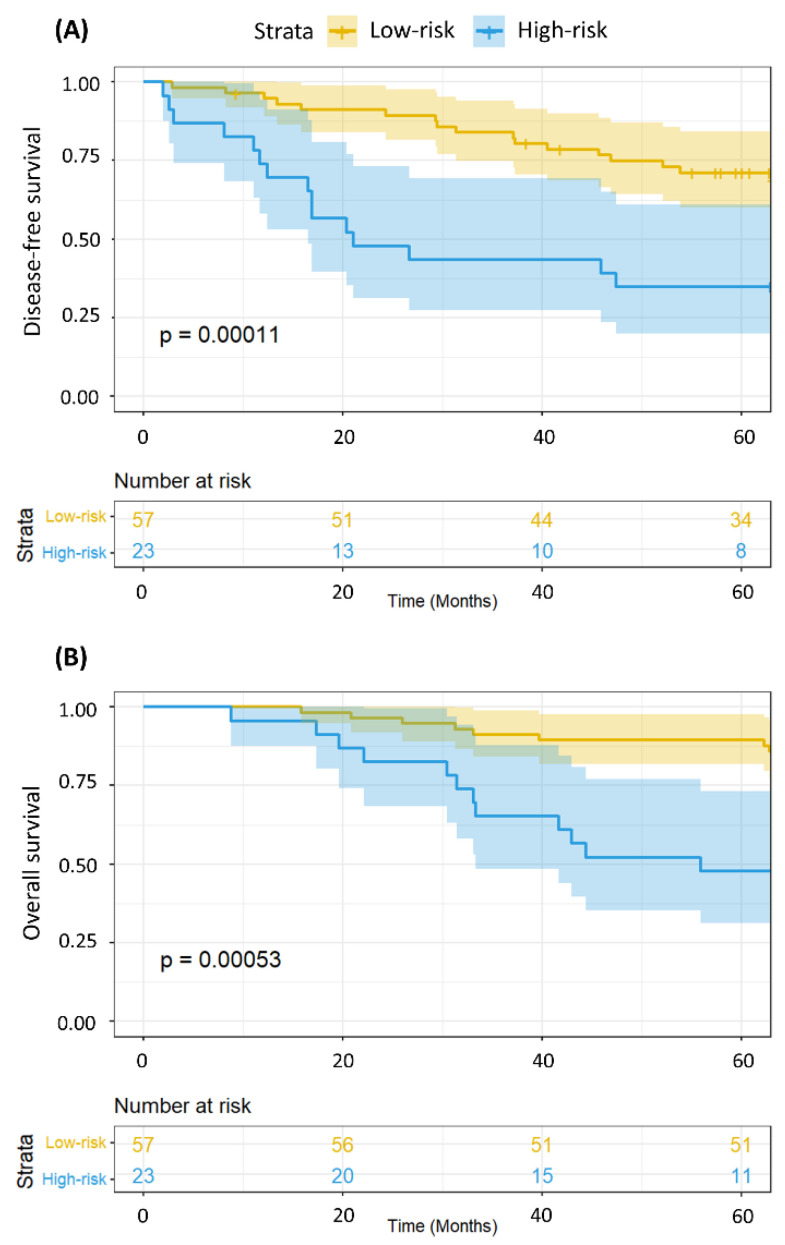
Kaplan-Meier representation of disease-free survival (**A**) and overall survival (**B**) according to MRSS risk strata: low-risk (yellow); high-risk (blue).

To assess whether the MRSS could provide survival information regarding not only disease-free survival but also overall survival, we applied MRSS stratification to a Kaplan-Meier survival representation. Figure 1 shows the Kaplan-Meier representation of disease-free (A) and overall (B) survival of the 80 breast cancer patients in relation to the metabolomics-derived risk MRSS strata (low- and high-risk).

**Figure 2:**
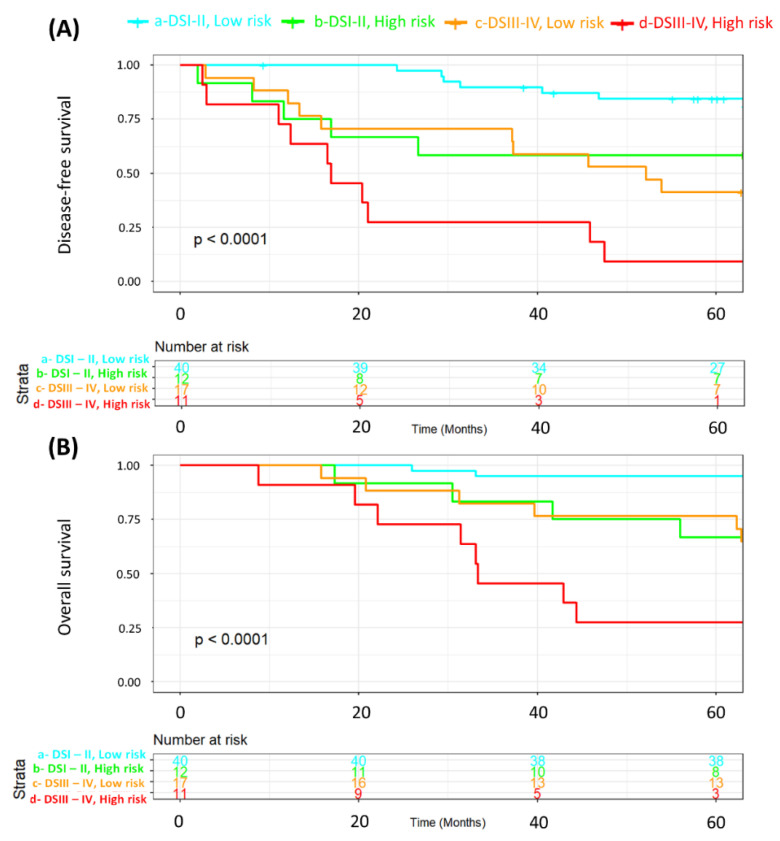
Kaplan-Meier representation of disease-free survival (**A**) and overall survival (**B**) according to MRSS risk strata (low and high) and disease stage. The survival curves for patients with disease stages I–II and low risk are depicted in cyan, whereas those for patients with disease stages I–II and high risk are depicted in green. For patients with disease stages III–IV, the low-risk stratum is depicted in orange, whereas the high-risk stratum is depicted in red.

**Figure 3:**
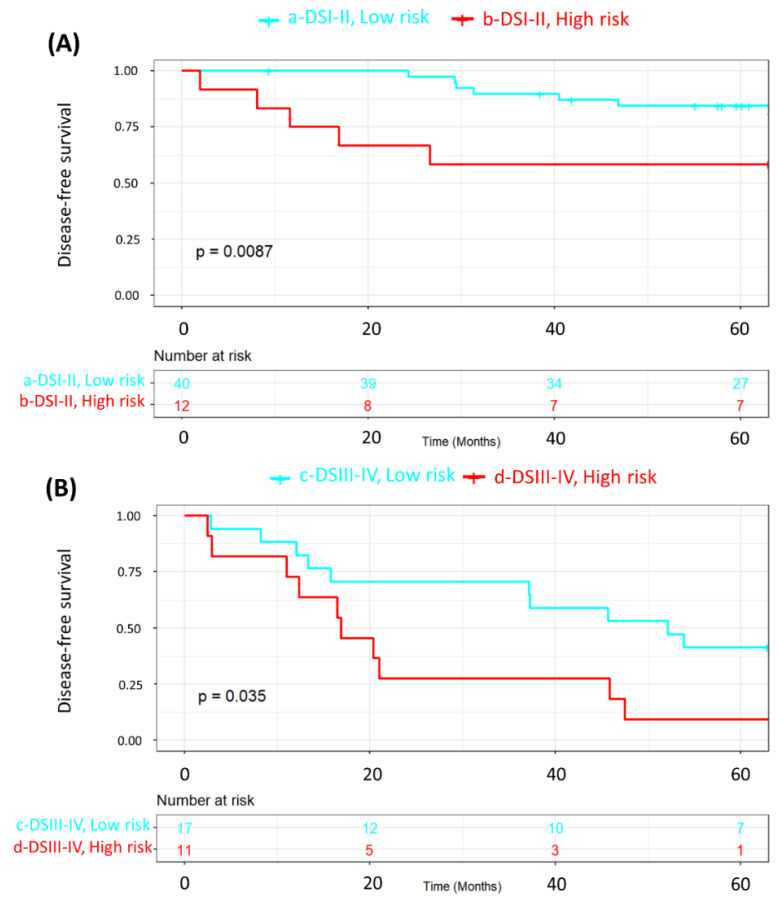
Kaplan-Meier representation of disease-free survival according to MRSS risk strata (low-risk, cyan; high-risk, red) in patients with disease stages I–II (**A**) and disease stages III–IV (**B**).

**Figure 4:**
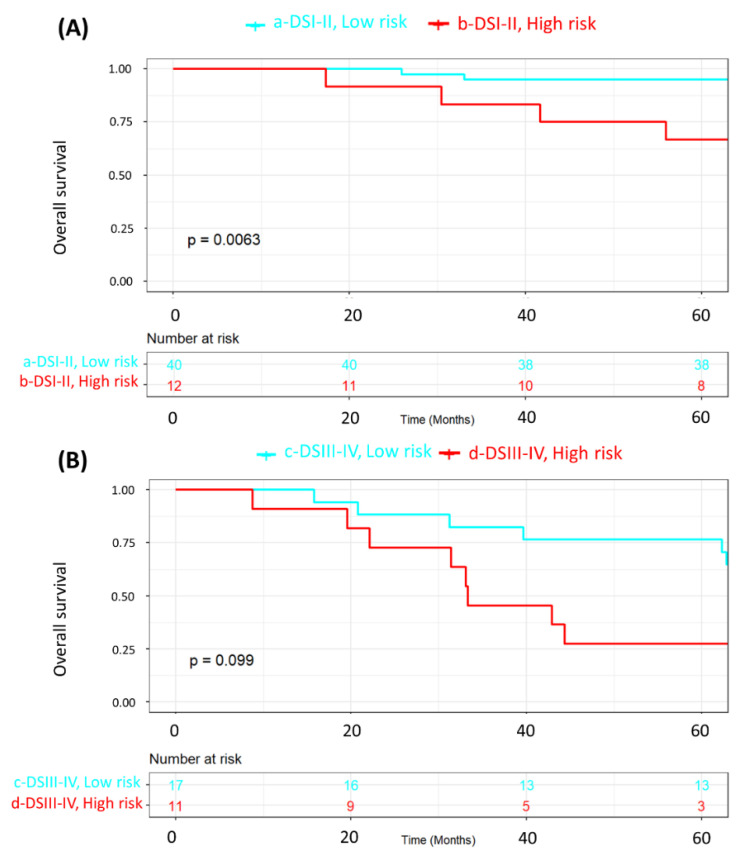
Kaplan-Meier representation of overall survival according to MRSS risk strata (low-risk, cyan; high-risk, red) in patients with disease stages I–II (**A**) and disease stages III–IV (**B**).

We also wanted to ascertain whether MRSS survival stratification was still valid in the subgroups of patients according to disease stage since staging is the most important standalone risk factor for disease relapse and death in breast cancer patients. Accordingly, Figure 2 combines low- and high-risk strata with disease stage (stages I–II vs. III–IV). Both Kaplan-Meier curves demonstrated significant differences in survival (*p* < 0.0001). Figure 3 is a subset of Figure 2, in which we compare the disease-free survival of patients with either stage I–II (A) or stage III–IV (B) disease according to the MRSS-risk strata. For patients with stage I–II disease (Figure 3A), disease-free survival was significantly (*p* = 0.0087) poorer for patients in the high-risk group, and the same phenomenon was shown for patients with advanced (stages III–IV) disease (*p* = 0.035, Figure 3B). Figure 4 shows the same analysis results as in Figure 3 but for overall survival. Overall survival was poorer in the high-risk strata for patients with disease stages I–II (*p* = 0.0063; Figure 4A) but not for patients with disease stages III–IV (*p* = 0.099; Figure 4B). These analyses demonstrate that MRSS survival information is valid regardless of disease stage and whether we are looking at disease-free or overall survival.

### 2.4. Metabolomics Data Can Provide Additional Survival Information Even after Adjustment for Disease Stage and Patient Age

Although our previous Kaplan-Meier analyses clearly demonstrated that the MRSS could stratify the disease-free survival and overall survival of patients with either early (stages I–II) or advanced (stages III–IV) breast cancer, these were bivariate analyses. To obtain a more robust assessment of MRSS potential as a prognostic tool, we opted to evaluate whether MRSS risk stratification contributed to OS prognosis even after multivariate adjustments for patient age and disease stage at diagnosis. As shown in Table 4, in the disease-free model, patients with a high-risk MRSS had a hazard ratio (HR) of 3.42 (95% CI 1.51–7.74; *p* = 0.003), indicating that the metabolomics data provided additional disease-free information. A similar trend was observed for overall survival, with patients at high risk according to metabolomics exhibiting an adjusted HR of 3.34 (95% CI 1.64–6.80; *p* < 0.001). It is worth mentioning that the molecular features of the tumor were considered to determine the MRSS (please refer to the methods section).

## 3. Discussion

Our study findings offer valuable insights into the associations between variations in serum metabolite abundances before and after neoadjuvant chemotherapy (NACT) and the clinical outcomes of women with breast cancer (BC) associated with patient survival. Using metabolomics data and steroid receptor and HER-2 data, we developed a metabolite-related survival score (MRSS). Importantly, the prognostic value of the MRSS remained significant even after adjusting the survival analyses for well-known prognostic factors such as age and disease stage. This effectively stratified patients into low- and high-risk disease relapse or progression groups, underscoring the potential of metabolomics as a tool for identifying patients at greater risk of disease relapse. Our analyses revealed that increases in histidine and lactate before and after NACT were associated with improved disease-free survival, whereas increases in serine and taurine abundances were associated with worse disease-free survival. Importantly, our study proposes a mathematical approach to resolve the collinearity problem often cited as an impediment to the utilization of metabolomics data in survival analyses [14].

Metabolomics holds promise for identifying disease markers and novel therapeutic targets, positioning it as a crucial component of precision medicine [15]. Studies on cancer metabolism have revealed how cancer cells undergo metabolic changes to support tumor growth. These changes are influenced by oncogenes, tumor suppressor genes, and the tumor microenvironment [16]. In the field of breast cancer research, studies have focused not only on identifying metabolic alterations that may contribute to cancer development [17,18,19,20,21] but also on assessing the response of patients to treatment and their prognosis [5,8,11,13,22,23,24].

To our knowledge, the most comparable study is Debik, et al. (2019), who utilized NMR to evaluate the systemic metabolic effects of NACT in patients with large primary breast cancers. However, that study did not find an association between serum changes and five-year survival [5]. In contrast, our study demonstrated a significant association between NACT-induced metabolic changes and patient survival. We believe that our mathematical approach may have contributed to revealing the relationship between metabolites and survival by mitigating collinearity within the CPH-derived coefficients via the VIF and deriving a score utilizing maximally selected rank statistics based on time-to-event outcomes. Interestingly, Debik and colleagues (2022) published a review article stressing the challenges posed by collinearity for the use of metabolomics data in survival analyses [14]. In the present study, we propose a methodology to address the mathematical and analytical problems listed in their review.

It is important to examine some literature data regarding the metabolites found to be associated with breast cancer survival in our analysis. Recently, a review on NMR and its relationship with breast cancer extensively surveyed the literature, exploring the connection between metabolites found in tissue, blood, and urine at distinct stages of breast cancer development [25]. Although the primary focus of that review was not patients undergoing NACT, some of the summarized data provide significant insights to contextualize our findings. For instance, a multicenter study analyzed serum samples from 590 patients with early breast cancer and 109 patients with metastatic breast cancer over a 5-year period. Among the early breast cancer patients, elevated levels of lactate and histidine were associated with a greater risk of relapse. However, as those authors pointed out, lactate is the most sensitive marker for sample degradation, and the fact that samples had been collected from different clinical sites using different operating and storing procedures might have influenced the results, leading the authors to remove lactate from the data matrix. Additionally, in comparison to early breast cancer patients, those with metastatic breast cancer exhibited elevated levels of lactate [26].

Histidine, an essential amino acid, is required for the synthesis of proteins and serves a critical role as a precursor for carnosine, a compound with antioxidant capabilities potentially inhibiting tumor growth and maintaining protein equilibrium during the aging process [27,28,29]. Recent findings indicate that the sensitivity of cancer cells to methotrexate—an anticancer treatment—can be influenced by both histidine catabolism and intake [30]. However, it is important to consider that none of the patients included in the present analysis received methotrexate as a component of their NACT. Notably, high lactate levels were not only linked to an unfavorable prognosis in BC tissue [31] but also noted in patients receiving NACT [5,32,33].

It is important to emphasize that our study focused on the changes in metabolite abundance from before to after NACT and on how much such abundance variation is associated with disease-free survival. Thus, metabolites for which increases in abundance from before to after NACT are associated with improved survival (lactate, histidine) may either play a role in metabolic anticancer mechanisms or be associated with improved effects of NACT drugs. Conversely, the reverse may be true for the metabolites for which increases in serum concentrations from before to after NACT were associated with poorer survival (serine and taurine). These considerations are necessary when interpreting our findings vis-à-vis the conclusions of other studies that evaluated such metabolites in the context of carcinogenic processes or even their associations with disease prognosis and response to treatments.

A key distinction between our study and previous research is that we focused on the changes in metabolite abundance from before to after neoadjuvant chemotherapy (NACT) and how these changes correlate with disease-free survival. For instance, metabolites like lactate and histidine, which increased in abundance following NACT, were associated with improved survival. This suggests that they may play a role in metabolic anticancer mechanisms or indicate a positive response to NACT. Conversely, increases in metabolites such as serine and taurine were linked to poorer survival outcomes. Our study is the first to integrate standard survival analysis (using Cox proportional hazards models) with metabolomics data, overcoming the issue of collinearity through a novel VIF approach. This approach allowed us to derive meaningful survival coefficients, unlike previous studies that assessed metabolite concentrations with survival using binary outcomes like “relapse/no-relapse” or “deceased/alive” at a specific point in time. By calculating survival probabilities over a follow-up period, we provide a more nuanced analysis.

To ensure that our findings can be adequately compared with those from other studies, it is crucial that similar statistical methodologies are employed. As our study pioneers the combination of standard survival analyses with metabolomics while addressing collinearity, we anticipate that future research will build on our methods to further explore these associations.

Serine is a nonessential amino acid that plays a vital role in sustaining several metabolic processes essential for the growth and survival of rapidly proliferating cells [34]. Previous research has suggested a connection between serine concentrations and the progression of cancer [35,36,37]. The serine synthesis pathway has been identified as essential in breast cancer [38]. Furthermore, BC patients exclusively treated with endocrine therapy exhibited decreased concentrations of serine [39]. It is important to mention that serine and glycine have a close metabolic relationship, with glycine being an amino acid produced from serine. Glycine is involved in collagen synthesis, is highly dysregulated in cancer, and plays both protumorigenic and antitumorigenic roles [40]. The increased concentration of such metabolites in the tumor microenvironment may explain their concentration fluctuations in the serum from before to after NACT. It is important to highlight that serine is an immunosuppressive metabolite that inhibits macrophage and neutrophil function. Moreover, the de novo serine synthesis pathway activity in macrophages is necessary for interleukin production, which induces a phenotypic switch to immunosuppressive PD-L1-expressing macrophages [35]. Thus, the overproduction of serine by cancer cells may promote the survival of nontransformed neighboring cells that create a protective niche for tumor maintenance [35].

Finally, our study showed that an increase in the serum taurine concentration from before to after NACT was associated with worse disease-free survival. Taurine is a nonessential amino acid that is abundant in many mammalian tissues. It has antioxidant functions, protects cells from oxidative stress, and plays an important role in the nervous system [41,42,43]. It has been demonstrated that taurine induces the apoptosis of breast cancer cells by regulating apoptosis-related proteins of the mitochondria [43]. Another study suggested that taurine exerts anti-breast cancer effects by regulating metabolism [44]. Furthermore, reduced levels of taurine were detected in urine samples from breast cancer patients in comparison to those from healthy controls [45,46,47]. Thus, an increase in the serum abundance of taurine may be a marker of a metabolic response against progressing cancer in the event of a failed response to NACT.

One intriguing aspect of our study is that none of the examined patient characteristics, except for a fortuitous association with prior pregnancy or tumor features, were found to be associated with MRSS status. This suggests that the MRSS, or simply metabolomics data, may provide independent predictive value beyond classical prognostic factors, making it a promising adjunctive tool for risk stratification in breast cancer patients. The robust statistical significance highlights the clinical utility of metabolomics data in predicting patient outcomes and guiding treatment decisions.

While our study has provided important findings, we must acknowledge its limitations. Overall, the small sample size and single-institution setting may have influenced the statistical power and generalizability of our results. Therefore, validation in larger, multicenter studies is warranted to confirm the robustness and applicability of our findings. Nonetheless, our preliminary observations offer valuable insights into the potential significance of specific changes in serum metabolite concentrations before and after NACT and the associations of these changes with breast cancer outcomes. Importantly, our study provides a roadmap for the use of metabolomic data in survival models.

## 4. Materials and Methods

### 4.1. Patient Selection, Accrual, and Sample Processing

The patient selection and accrual process, the clinical, histopathological, and immunohistochemical diagnosis of breast cancer, and untargeted nuclear magnetic resonance (NMR) metabolomic analysis of serum samples have been described elsewhere [13]. Briefly, the serum samples were thawed at room temperature before the analysis. Then, 400 µL of serum was slowly mixed with 200 µL of D_2_O (99.9% deuterium oxide with 0.03% of TSP) and transferred to 5 mm NMR tubes. The NMR experiments were performed at 298 K on Varian Inova^®^ NMR spectrometer (Agilent Technologies^®^ Inc., Santa Clara, CA, USA) in the Brazilian Biosciences National Laboratory (Brazilian Center for Research in Energy and Materials, CNPEM, Campinas, SP, Brazil), operating in Larmor frequency of 599.887 MHz equipped with triple resonance cryoprobe.

Data related to preprocessing, spectral phase, and baseline corrections as well as the identification and quantification of relative concentrations (in mM) of metabolites present in the samples were performed using the Chenomx NMR^®^ Suite 8.1 software (Chenomx^®^ Inc., Edmonton, AB, Canada) (Figure S2).

### 4.2. Clinical Follow-Up

**Figure 5:**
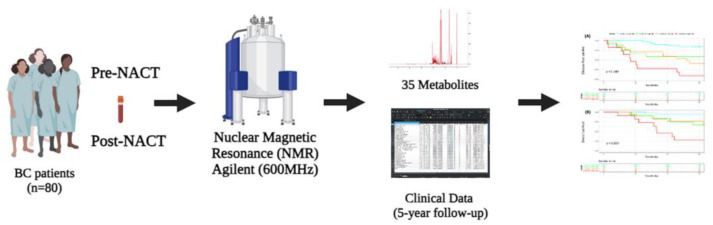
Schematic representation of the study design. Metabolomic and clinical data combined to predict patient survival following neoadjuvant chemotherapy.

In this study, we obtained complete follow-up data from 80 women who were diagnosed with invasive breast carcinoma between January 2017 and January 2019 and who underwent neoadjuvant chemotherapy (NACT) followed by surgery at the Women’s Hospital (Hospital da Mulher Prof. Dr. José Aristodemo Pinotti, Centro de Atenção Integral à Saúde da Mulher—CAISM) of the University of Campinas (UNICAMP). Patients were followed up through to December 2023. As previously described [13], serum samples were collected at two time points—before the first infusion of NACT and up to two months after the completion of NACT—with new aliquots of peripheral blood obtained (Figure 5). All samples were stored in CAISM’s biobank (CONEP 56, Brazil) according to the biobank’s protocol until processing for NMR analysis. Breast cancer diagnosis and NACT protocols were performed in accordance with institutional protocols [13].

All patients included in this study were regularly followed up at the hospital’s breast cancer clinics. The mean follow-up time was 63.15 ± 19.2 months, with an interquartile range of 12.4 months. Disease-free intervals were calculated by measuring the time between the first NACT session and the date of relapse diagnosis. Overall survival was calculated as the time elapsed between the start of NACT and the date of death. Follow-up consultations were conducted at specific intervals, including 21-day intervals during NACT, 15-day intervals during the first month after surgery, and 3-month intervals six months after completing therapy, followed by 6-month intervals thereafter. 

### 4.3. Metabolite Normalization

We normalized the metabolite quantification of relative concentrations (mmol L^−1^) by sum (metabolite concentration divided by the sum of all metabolite concentrations for that patient) and applied logarithmic (base e) conversion to the normalized metabolite concentrations. The same normalization and logarithmic conversion procedures were applied to the relative concentrations of metabolites in samples collected either before or after NACT.

### 4.4. Metabolite Differential Fold-Changes

We calculated the fold change for each metabolite by considering the variation in the relative normalized concentration before and after NACT. The fold-change values were calculated using paired analysis, where the ratio between paired samples (metabolite concentration after NACT divided by the metabolite concentration before NACT) was computed to determine the fold-change for each pair (Figure S3). The mean of the fold changes was then calculated, indicating whether there was an increase or decrease in the relative concentration of a metabolite after NACT (Figure S1).

### 4.5. Metabolite Contribution Using Multivariate Cox Proportional Hazards Selected by the Variation Inflation Factor (VIF)

For the disease-free survival analyses, we employed multivariate Cox proportional hazards (CPH) models. The models were constructed using the full set of 35 studied metabolites, steroid (estrogen, progesterone) receptor status, and HER-2 status as covariates. To account for potential multicollinearity effects in the quantified metabolite concentration data, we calculated the variation inflation factor (VIF) for the metabolites included in the CPH models [48]. The variable with the highest VIF was recursively removed, and the CPH model was rerun iteratively until all remaining variables had a VIF < 3 (Table 1). Table S1 illustrates the relative abundance of each metabolite before and after NACT, the fold change from before to after NACT, and the unadjusted and adjusted *p*-values for the survival analyses using the fold changes for each metabolite as survival factors. *p*-values refer to the individual survival contribution attributable to the fold-change for each metabolite. These individual analyses suffer from the collinearity issue found in metabolomics data; this problem was addressed using our VIF approach. 

### 4.6. Metabolite-Related Survival Score (MRSS)

To evaluate whether the variation in metabolite concentration from before to after NACT (fold change) could discern patient survival probabilities, we calculated a metabolite-related survival score (MRSS). We set the significance threshold at *p* < 0.10 to select the metabolites used in constructing the MRSS: histidine (*p* = 0.04), lactate (*p* = 0.07), serine (*p* = 0.01), and taurine (*p* = 0.03). The MRSS was determined by summing the multiplication of the CPH-derived correlation coefficients for the four-metabolite fold changes significantly associated with disease-free survival with their respective normalized and logarithmically converted concentration ratios (fold change in metabolite abundance after/before NACT). Using maximally selected rank statistics based on time-to-event outcomes [49], we divided the patient cohorts into two groups with the most significant differences between them, creating low- and high-risk relapse cohorts based on the MRSS.

Furthermore, we analyzed the associations between key patient characteristics (Table 2) and their respective tumor features (Table 3) in the MRSS-derived cohort groups (low- and high-risk patients) using chi-square statistics and Fisher’s *t* tests, where appropriate. Kaplan–Meier survival curves and log-rank tests were used to assess disease-free survival based on the MRSS (Figure 1). Next, we examined disease-free survival (DFS) and overall survival (OS) as related to the MRSS in patients with disease stages I–II and stages III–IV (Figure 2, Figure 3 and Figure 4).

Finally, to assess whether the MRSS could provide prognostic information beyond the main classical prognostic factors (patient age and disease stage) in a multivariate environment, we performed multivariate Cox proportional hazards analysis for disease-free survival and OS, including these classical prognostic factors and MRSS as covariates (Table 4). It is worth mentioning that steroid receptor and HER-2 status had already been used to adjust the survival models from which MRSS-contributing metabolite coefficients were derived. All statistical calculations were performed using R, a language and environment for statistical computing [50].

## Figures and Tables

**Table 1:** Variance inflation factor (VIF)-guided multivariate Cox proportional hazards coefficients of disease-free survival as related to the fold change* of metabolite abundances.

		Disease-Free Survival ^†^		
Metabolite	VIF	HR	(CI 95%)	*p*-Value
Alanine	2.88	2.88	(0.44–18.83)	0.27
Arginine	2.92	1.28	(0.31–5.23)	0.73
Ascorbate	2.09	0.85	(0.46–1.59)	0.62
Asparagine	2.21	0.97	(0.41–2.33)	0.95
Aspartate	2.24	1.51	(0.69–3.30)	0.30
Betaine	>3	-	-	-
Carnitine	>3	-	-	-
Choline	2.77	0.47	(0.14–1.56)	0.22
Citrate	2.32	1.66	(0.40–6.90)	0.49
Creatine	1.89	0.59	(0.26–1.32)	0.20
Creatinine	2.07	3.81	(0.65–22.12)	0.14
Formate	>3	-	-	-
Glucose	>3	-	-	-
Glutamate	2.77	1.65	(0.41–6.59)	0.48
Glutamine	2.20	2.37	(0.30–18.75)	0.41
Glycerol	2.08	0.58	(0.25–1.32)	0.19
Glycine	>3	-	-	-
Histidine	2.75	0.32	(0.11–0.95)	0.04
Isoleucine	>3	-	-	-
Lactate	2.48	0.32	(0.09–1.11)	0.07
Leucine	>3	-	-	-
Lysine	>3	-	-	-
Methionine	>3	-	-	-
myo-Inositol	2.34	1.27	(0.36–4.40)	0.71
N,N-Dimethylglycine	2.05	0.53	(0.18–1.51)	0.23
Pantothenate	2.83	0.84	(0.26–2.74)	0.77
Phenylalanine	2.24	0.97	(0.49–1.92)	0.94
Proline	2.26	0.58	(0.17–2.01)	0.39
Serine	2.81	5.06	(1.45–17.70)	0.01
sn-Glycero-3-phosphocholine	2.76	2.89	(0.47–17.81)	0.25
Taurine	1.63	2.51	(1.07–5.91)	0.03
Threonine	>3	-	-	-
Tyrosine	>3	-	-	-
Urea	>3	-	-	-
Valine	>3	-	-	-
HER-2	2.17	0.80	(0.28–2.31)	0.68
Hormonal Receptor	1.59	1.45	(0.53–3.96)	0.47

VIF: Variance inflation factor. HR: hazard ratio. CI: Confidence interval. * Fold change: ratio of metabolite abundances after/before neoadjuvant chemotherapy (NACT). ^†^ HR and *p*-values not calculated for metabolites with VIF > 3.

**Table 2:** Key patient characteristics related to metabolomics-derived risk strata (MRSS).

Characteristic		*n* (%)	Low Risk *n* = 57 (71%)	High Risk *n* = 23 (29%)	*p*-Value
Age (years)	≥50	46 (57.0)	34 (59.6)	12 (52.2)	0.72
	<50	34 (43.0)	23 (40.4)	11 (47.8)	
Disease Stage	I	4 (5.0)	3 (5.3)	1 (4.3)	0.46
	II	48 (60.0)	37 (64.9)	11 (47.8)	
	III	22 (27.5)	13 (22.8)	9 (39.1)	
	IV	6 (7.5)	4 (7.0)	2 (8.7)	
Race	Caucasian	69 (86.3)	48 (84.2)	21 (91.3)	0.63
	Noncaucasian	11 (13.8)	9 (15.8)	2 (8.7)	
Age of menarche (years)	>12	42 (52.5)	30 (52.6)	12 (52.2)	1.00
	≤12	38 (47.5)	27 (47.4)	11 (47.8)	
Menopause	No	36 (45.0)	24 (42.1)	12 (52.2)	0.57
	Yes	44 (55.0)	33 (57.9)	11 (47.8)	
Hormone replacement therapy	No	68 (85.0)	48 (84.2)	20 (87.0)	1.00
	Yes	12 (15.0)	9 (15.8)	3 (13.0)	
Previous pregnancy	Yes	73 (91.3)	55 (96.5)	18 (78.3)	0.03
	No	7 (8.7)	2 (3.5)	5 (21.7)	
Lactation *	Yes	63 (78.8)	46 (80.7)	17 (73.9)	0.71
	No	17 (21.2)	11 (19.3)	6 (26.1)	
Smoking	Yes	17 (21.2)	13 (22.8)	4 (17.4)	0.81
	No	63 (78.8)	44 (77.2)	19 (82.6)	
BMI categories	Normal weight	24 (30.0)	14 (24.6)	10 (43.5)	0.21
	Overweight	21 (26.3)	17 (29.8)	4 (17.4)	
	Obese	35 (43.7)	26 (45.6)	9 (39.1)	
Diabetes	No	71 (88.7)	50 (87.7)	21 (91.3)	0.94
	Yes	9 (11.3)	7 (12.3)	2 (8.7)	
Family history of breast or ovarian cancer	No	59 (73.8)	41 (71.9)	18 (78.3)	0.76
	Yes	21 (26.2)	16 (28.1)	5 (21.7)	
Relapse/progression	No	47 (58.7)	40 (70.2)	7 (30.4)	0.002
	Yes	33 (42.3)	17 (29.3)	16 (69.6)	
Death	No	55 (68.7)	45 (78.9)	10 (43.5)	0.004
	Yes	25 (32.3)	12 (21.1)	13 (56.3)	
Final status by the end of follow-up	Alive without disease	47 (58.8)	40 (70.2)	7 (30.4)	0.003
	Alive with disease	8 (10.0)	5 (8.8)	3 (13.0)	
	Deceased	25 (31.2)	12 (21.1)	13 (56.5)	

* Statistical significance based only on available data. MRSS: metabolite-related survival score.

**Table 3:** Main tumor features related to patient outcomes at the end of the follow-up.

Characteristic		*n* (%)	Low Risk *n* = 57 (71%)	High Risk *n* = 23 (29%)	*p*-Value
Histological grade	1/2	39 (48.75)	29 (50.9)	10 (43.5)	0.72
	3	41 (51.25)	28 (49.1)	13 (56.5)	
Ki67 (mean/SD)	Below 40%	35 (43.7)	27 (47.4)	8 (34.8)	0.44
	40% or higher	45 (57.5)	30 (52.6)	15 (65.2)	
HER-2	Negative	46 (57.5)	30 (52.6)	16 (69.6)	0.25
	Positive *	34 (42.5)	27 (47.4)	7 (30.4)	
Tumor size	T1/T2	53 (66.25)	40 (70.2)	13 (56.5)	0.36
	T3/T4	27 (33.75)	17 (29.8)	10 (43.5)	
Regional lymph node	N0	33 (41.25)	25 (43.9)	8 (34.8)	0.62
	N1 or higher	47 (58.75)	32 (56.1)	15 (65.2)	
Metastasis	M0	74 (92.5)	53 (93.0)	21 (91.3)	1.00
	M1	6 (7.5)	4 (7.0)	2 (8.7)	
Hormonal Receptor	Negative	21 (26.25)	13 (22.8)	8 (34.8)	0.41
	Positive	59 (73.75)	44 (77.2)	15 (65.2)	

SD: standard deviation. * Positive HER-2 status determined using immunohistochemistry and fluorescent in situ hybridization.

**Table 4:** Multivariate analysis of disease-free survival and overall survival as related to age, disease stage, and MRSS (Metabolite-Related Survival Score).

		Disease-Free Survival			Overall Survival		
Factor	*n* (%)	HR	(95%CI)	Adjusted *p*-Value *	HR	(95%CI)	Adjusted *p*-Value *
Age (years)							
≥50	46 (57%)	Ref.			Ref.		
<50	34 (43%)	0.51	(0.21–1.25)	0.15	0.67	(0.31–1.41)	0.29
Disease stage							
I–II	52 (65%)	Ref.			Ref.		
III–IV	28 (35%)	5.88	(2.32–14.88)	<0.001	4.49	(2.08–9.69)	<0.001
MRSS							
Low	57 (71%)	Ref.			Ref.		
High	23 (29%)	3.42	(1.51–7.74)	0.003	3.34	(1.64–6.80)	<0.001

Ref.: referential. HR: hazard ratio. CI: Confidence interval. * Cox Proportional Hazards Model using as covariates age, disease stage, and MRSS.

## Data Availability

The datasets generated during the current study are available from the corresponding author upon reasonable request.

## Supplementary Materials

The following supporting information can be downloaded at: https://www.mdpi.com/article/10.3390/ijms25168639/s1.

## References

[1] Sung H., Ferlay J., Siegel R.L., Laversanne M., Soerjomataram I., Jemal A., Bray F. (2021). Global Cancer Statistics 2020: GLOBOCAN Estimates of Incidence and Mortality Worldwide for 36 Cancers in 185 Countries. CA. Cancer J. Clin..

[2] Haddad T.C., Goetz M.P. (2015). Landscape of Neoadjuvant Therapy for Breast Cancer. Ann. Surg. Oncol..

[3] Denkert C., von Minckwitz G., Darb-Esfahani S., Lederer B., Heppner B.I., Weber K.E., Budczies J., Huober J., Klauschen F., Furlanetto J. (2018). Tumour-Infiltrating Lymphocytes and Prognosis in Different Subtypes of Breast Cancer: A Pooled Analysis of 3771 Patients Treated with Neoadjuvant Therapy. Lancet. Oncol..

[4] Kim R., Kin T. (2021). Clinical Perspectives in Addressing Unsolved Issues in (Neo)Adjuvant Therapy for Primary Breast Cancer. Cancers.

[5] Debik J., Euceda L.R., Lundgren S., Gythfeldt H.V.D.L., Garred Ø., Borgen E., Engebraaten O., Bathen T.F., Giskeødegård G.F. (2019). Assessing Treatment Response and Prognosis by Serum and Tissue Metabolomics in Breast Cancer Patients. J. Proteome Res..

[6] Lin X., Xu R., Mao S., Zhang Y., Dai Y., Guo Q., Song X., Zhang Q., Li L., Chen Q. (2019). Metabolic Biomarker Signature for Predicting the Effect of Neoadjuvant Chemotherapy of Breast Cancer. Ann. Transl. Med..

[7] Salvador-Coloma C., Santaballa A., Sanmartín E., Calvo D., García A., Hervás D., Cordón L., Quintas G., Ripoll F., Panadero J. (2020). Immunosuppressive Profiles in Liquid Biopsy at Diagnosis Predict Response to Neoadjuvant Chemotherapy in Triple-Negative Breast Cancer. Eur. J. Cancer.

[8] He X., Gu J., Zou D., Yang H., Zhang Y., Ding Y., Teng L. (2021). NMR-Based Metabolomics Analysis Predicts Response to Neoadjuvant Chemotherapy for Triple-Negative Breast Cancer. Front. Mol. Biosci..

[9] Díaz C., González-Olmedo C., Díaz-Beltrán L., Camacho J., Mena García P., Martín-Blázquez A., Fernández-Navarro M., Ortega-Granados A.L., Gálvez-Montosa F., Marchal J.A. (2022). Predicting Dynamic Response to Neoadjuvant Chemotherapy in Breast Cancer: A Novel Metabolomics Approach. Mol. Oncol..

[10] Zapater-Moros A., Díaz-Beltrán L., Gámez-Pozo A., Trilla-Fuertes L., Lumbreras-Herrera M.I., López-Camacho E., González-Olmedo C., Espinosa E., Zamora P., Sánchez-Rovira P. (2023). Metabolomics Unravels Subtype-Specific Characteristics Related to Neoadjuvant Therapy Response in Breast Cancer Patients. Metabolomics.

[11] Vignoli A., Ghini V., Meoni G., Licari C., Takis P.G., Tenori L., Turano P., Luchinat C. (2019). High-Throughput Metabolomics by 1D NMR. Angew. Chem. Int. Ed. Engl..

[12] Hanahan D., Weinberg R.A. (2011). Hallmarks of Cancer: The next Generation. Cell.

[13] Cardoso M.R., Silva A.A.R., Talarico M.C.R., Sanches P.H.G., Sforça M.L., Rocco S.A., Rezende L.M., Quintero M., Costa T.B.B.C., Viana L.R. (2022). Metabolomics by NMR Combined with Machine Learning to Predict Neoadjuvant Chemotherapy Response for Breast Cancer. Cancers.

[14] Debik J., Sangermani M., Wang F., Madssen T.S., Giskeødegård G.F. (2022). Multivariate Analysis of NMR-Based Metabolomic Data. NMR Biomed..

[15] Clish C.B. (2015). Metabolomics: An Emerging but Powerful Tool for Precision Medicine. Cold Spring Harb. Mol. Case Stud..

[16] Pavlova N.N., Thompson C.B. (2016). The Emerging Hallmarks of Cancer Metabolism. Cell Metab..

[17] Suman S., Sharma R.K., Kumar V., Sinha N., Shukla Y. (2018). Metabolic Fingerprinting in Breast Cancer Stages through (1)H NMR Spectroscopy-Based Metabolomic Analysis of Plasma. J. Pharm. Biomed. Anal..

[18] Gumà J., Adriá-Cebrián J., Ruiz-Aguado B., Albacar C., Girona J., Rodríguez-Calvo R., Martínez-Micaelo N., Lam E.W.F., Masana L., Guaita-Esteruelas S. (2021). Altered Serum Metabolic Profile Assessed by Advanced ^1^H-NMR in Breast Cancer Patients. Cancers.

[19] Lécuyer L., Victor Bala A., Deschasaux M., Bouchemal N., Nawfal Triba M., Vasson M.-P., Rossary A., Demidem A., Galan P., Hercberg S. (2018). NMR Metabolomic Signatures Reveal Predictive Plasma Metabolites Associated with Long-Term Risk of Developing Breast Cancer. Int. J. Epidemiol..

[20] Corona G., Di Gregorio E., Vignoli A., Muraro E., Steffan A., Miolo G. (2021). ^1^H-NMR Plasma Lipoproteins Profile Analysis Reveals Lipid Metabolism Alterations in HER2-Positive Breast Cancer Patients. Cancers.

[21] Jobard E., Dossus L., Baglietto L., Fornili M., Lécuyer L., Mancini F.R., Gunter M.J., Trédan O., Boutron-Ruault M.-C., Elena-Herrmann B. (2021). Investigation of Circulating Metabolites Associated with Breast Cancer Risk by Untargeted Metabolomics: A Case-Control Study Nested within the French E3N Cohort. Br. J. Cancer.

[22] Asiago V.M., Alvarado L.Z., Shanaiah N., Gowda G.A.N., Owusu-Sarfo K., Ballas R.A., Raftery D. (2010). Early Detection of Recurrent Breast Cancer Using Metabolite Profiling. Cancer Res..

[23] Choi J.S., Baek H.-M., Kim S.S.I.S., Kim M.J., Youk J.H., Moon H.J., Kim E.-K., Han K.H., Kim D.-H., Kim S.S.I.S. (2012). HR-MAS MR Spectroscopy of Breast Cancer Tissue Obtained with Core Needle Biopsy: Correlation with Prognostic Factors. PLoS ONE.

[24] Zidi O., Souai N., Raies H., Ben Ayed F., Mezlini A., Mezrioui S., Tranchida F., Sabatier J.-M., Mosbah A., Cherif A. (2021). Fecal Metabolic Profiling of Breast Cancer Patients during Neoadjuvant Chemotherapy Reveals Potential Biomarkers. Molecules.

[25] Vignoli A., Risi E., McCartney A., Migliaccio I., Moretti E., Malorni L., Luchinat C., Biganzoli L., Tenori L. (2021). Precision Oncology via NMR-Based Metabolomics: A Review on Breast Cancer. Int. J. Mol. Sci..

[26] Hart C.D., Vignoli A., Tenori L., Uy G.L., Van To T., Adebamowo C., Hossain S.M., Biganzoli L., Risi E., Love R.R. (2017). Serum Metabolomic Profiles Identify ER-Positive Early Breast Cancer Patients at Increased Risk of Disease Recurrence in a Multicenter Population. Clin. Cancer Res..

[27] Barnes T., Bell K., DiSebastiano K.M., Vance V., Hanning R., Russell C., Dubin J.A., Bahl M., Califaretti N., Campbell C. (2014). Plasma Amino Acid Profiles of Breast Cancer Patients Early in the Trajectory of the Disease Differ from Healthy Comparison Groups. Appl. Physiol. Nutr. Metab..

[28] Brosnan M.E., Brosnan J.T. (2020). Histidine Metabolism and Function. J. Nutr..

[29] Moro J., Tomé D., Schmidely P., Demersay T.-C., Azzout-Marniche D. (2020). Histidine: A Systematic Review on Metabolism and Physiological Effects in Human and Different Animal Species. Nutrients.

[30] Kanarek N., Keys H.R., Cantor J.R., Lewis C.A., Chan S.H., Kunchok T., Abu-Remaileh M., Freinkman E., Schweitzer L.D., Sabatini D.M. (2018). Histidine Catabolism Is a Major Determinant of Methotrexate Sensitivity. Nature.

[31] Giskeødegård G.F., Lundgren S., Sitter B., Fjøsne H.E., Postma G., Buydens L.M.C., Gribbestad I.S., Bathen T.F. (2012). Lactate and Glycine-Potential MR Biomarkers of Prognosis in Estrogen Receptor-Positive Breast Cancers. NMR Biomed..

[32] Euceda L.R., Haukaas T.H., Giskeødegård G.F., Vettukattil R., Engel J., Silwal-Pandit L., Lundgren S., Borgen E., Garred Ø., Postma G. (2017). Evaluation of Metabolomic Changes during Neoadjuvant Chemotherapy Combined with Bevacizumab in Breast Cancer Using MR Spectroscopy. Metabolomics.

[33] Cao M.D., Giskeødegård G.F., Bathen T.F., Sitter B., Bofin A., Lønning P.E., Lundgren S., Gribbestad I.S. (2012). Prognostic Value of Metabolic Response in Breast Cancer Patients Receiving Neoadjuvant Chemotherapy. BMC Cancer.

[34] Yang M., Vousden K.H. (2016). Serine and One-Carbon Metabolism in Cancer. Nat. Rev. Cancer.

[35] Geeraerts S.L., Heylen E., De Keersmaecker K., Kampen K.R. (2021). The Ins and Outs of Serine and Glycine Metabolism in Cancer. Nat. Metab..

[36] Li A.M., Ye J. (2020). Reprogramming of Serine, Glycine and One-Carbon Metabolism in Cancer. Biochim. Biophys. Acta—Mol. Basis Dis..

[37] Reina-Campos M., Linares J.F., Duran A., Cordes T., L’Hermitte A., Badur M.G., Bhangoo M.S., Thorson P.K., Richards A., Rooslid T. (2019). Increased Serine and One-Carbon Pathway Metabolism by PKCλ/ι Deficiency Promotes Neuroendocrine Prostate Cancer. Cancer Cell.

[38] Possemato R., Marks K.M., Shaul Y.D., Pacold M.E., Kim D., Birsoy K., Sethumadhavan S., Woo H.-K., Jang H.G., Jha A.K. (2011). Functional Genomics Reveal That the Serine Synthesis Pathway Is Essential in Breast Cancer. Nature.

[39] Nees J., Schafferer S., Yuan B., Tang Q., Scheffler M., Hartkopf A., Golatta M., Schneeweiß A., Burwinkel B., Wallwiener M. (2022). How Previous Treatment Changes the Metabolomic Profile in Patients with Metastatic Breast Cancer. Arch. Gynecol. Obstet..

[40] Cox T.R. (2021). The Matrix in Cancer. Nat. Rev. Cancer.

[41] Matés J.M., Segura J.A., Alonso F.J., Márquez J. (2012). Oxidative Stress in Apoptosis and Cancer: An Update. Arch. Toxicol..

[42] Chen C., Xia S., He J., Lu G., Xie Z., Han H. (2019). Roles of Taurine in Cognitive Function of Physiology, Pathologies and Toxication. Life Sci..

[43] Zhang X., Lu H., Wang Y., Liu C., Zhu W., Zheng S., Wan F. (2015). Taurine Induces the Apoptosis of Breast Cancer Cells by Regulating Apoptosis-Related Proteins of Mitochondria. Int. J. Mol. Med..

[44] Chen W., Li Q., Hou R., Liang H., Zhang Y., Yang Y. (2022). An Integrated Metabonomics Study to Reveal the Inhibitory Effect and Metabolism Regulation of Taurine on Breast Cancer. J. Pharm. Biomed. Anal..

[45] Slupsky C.M., Steed H., Wells T.H., Dabbs K., Schepansky A., Capstick V., Faught W., Sawyer M.B. (2010). Urine Metabolite Analysis Offers Potential Early Diagnosis of Ovarian and Breast Cancers. Clin. Cancer Res. Off. J. Am. Assoc. Cancer Res..

[46] Silva C.L., Olival A., Perestrelo R., Silva P., Tomás H., Câmara J.S. (2019). Untargeted Urinary ^1^H NMR-Based Metabolomic Pattern as a Potential Platform in Breast Cancer Detection. Metabolites.

[47] Men Y., Li L., Zhang F., Kong X., Zhang W., Hao C., Wang G. (2020). Evaluation of Heavy Metals and Metabolites in the Urine of Patients with Breast Cancer. Oncol. Lett..

[48] Fox J., Monette G. (1992). Generalized Collinearity Diagnostics. J. Am. Stat. Assoc..

[49] Lausen B., Schumacher M. (1992). Maximally Selected Rank Statistics. Biometrics.

[50] Team R Core R: A Language and Environment for Statistical Computing.

