# Supplementary material for: Metabolomic Profiling of Breast Cancer Patients Undergoing Neoadjuvant Chemotherapy for Predicting Disease-Free and Overall Survival
Source: Int J Mol Sci. 2024 Aug 8;25(16):8639. doi: 10.3390/ijms25168639 (PMC11354796; doi:10.3390/ijms25168639)
Supplement: Supplementary file 1 [file ijms-25-08639-s001.zip › ijms-3101932-supplementary.pdf]

Metabolomic Profiling of Breast Cancer Patients Undergoing Neoadjuvant Chemotherapy for Predicting Disease-Free and Overall Survival

Maria Cecília Ramiro Talarico, Sophie Derchain, Lucas Ferreira da Silva, Maurício L. Sforça, Silvana A. Rocco, Marcella R. Cardoso, Luís Otávio Sarian

Supplementary Materials

List of Contents:

1. Supplementary Figures:

Figure S1 – Mean ratios of after NACT/before NACT normalized NMR serum metabolite abundance. Values > 0 indicate that the serum abundance of a particular metabolite was higher after NACT compared to before NACT. Values < 0 indicate the contrary.

Figure S2 – Representative NMR spectra from serum of women who underwent NACT.

Figure S3 – Box plot representation of the studied metabolites' fold changes (concentration after NACT/concentration before NACT).

2. Supplementary Table:

Table S1 – Average relative abundances of the serum metabolites detected by NMR according to response to NACT.

Supplementary Material

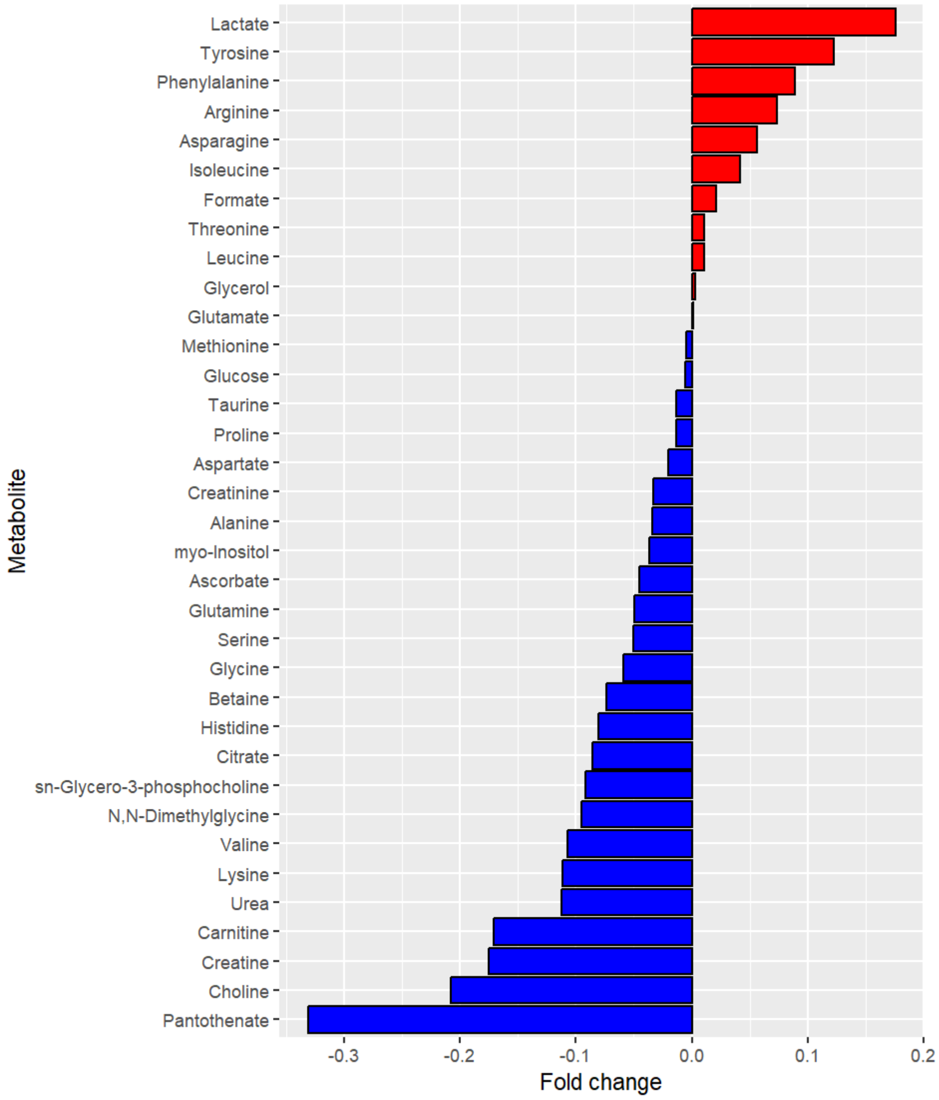

Figure S1 – Mean ratios of after NACT/before NACT normalized NMR serum metabolite abundance. Values > 0 indicate that the serum abundance of a particular metabolite was higher after NACT compared to before NACT. Values < 0 indicate the contrary.

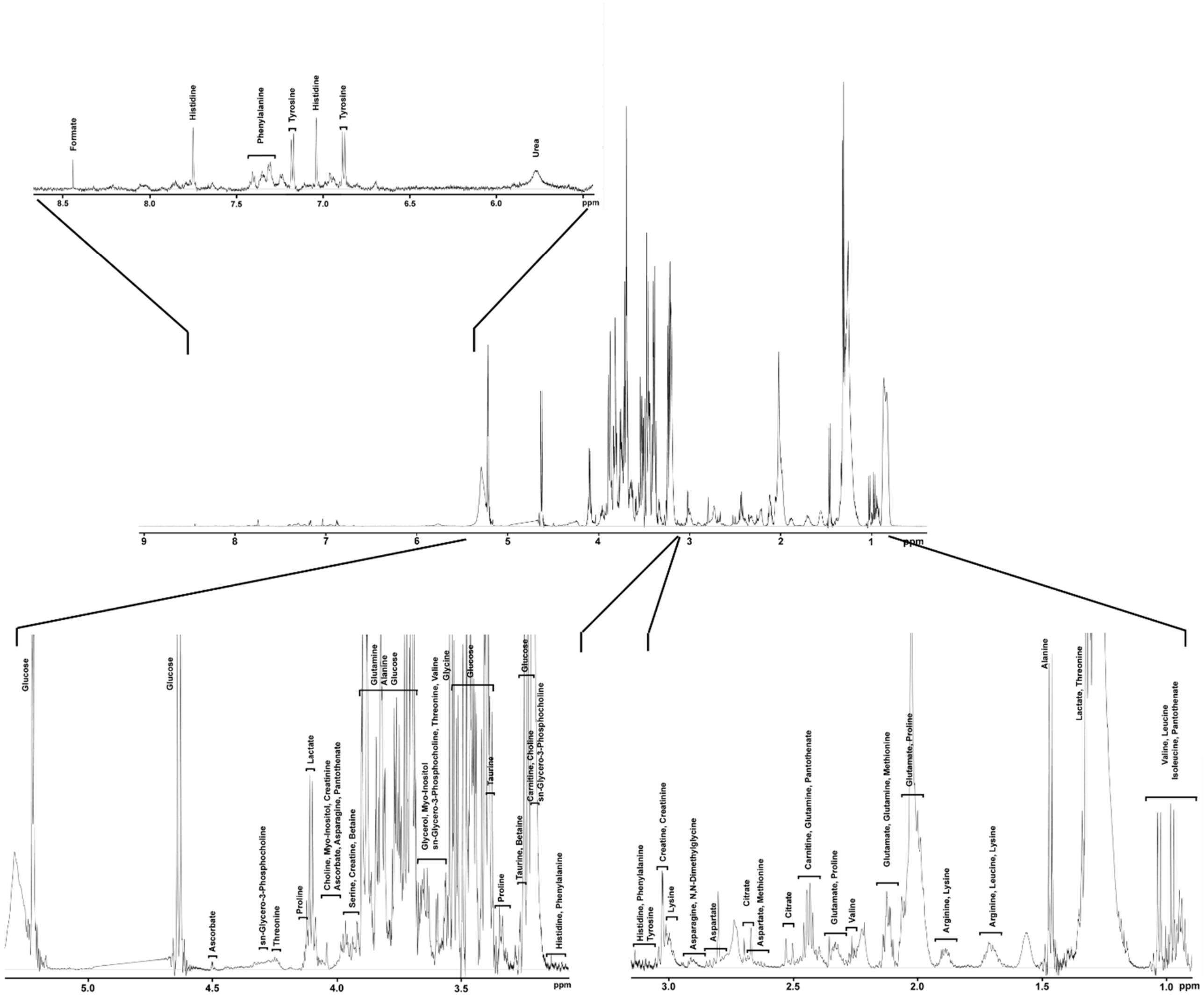

Figure S2 – Representative NMR spectra from serum of women who underwent NACT.

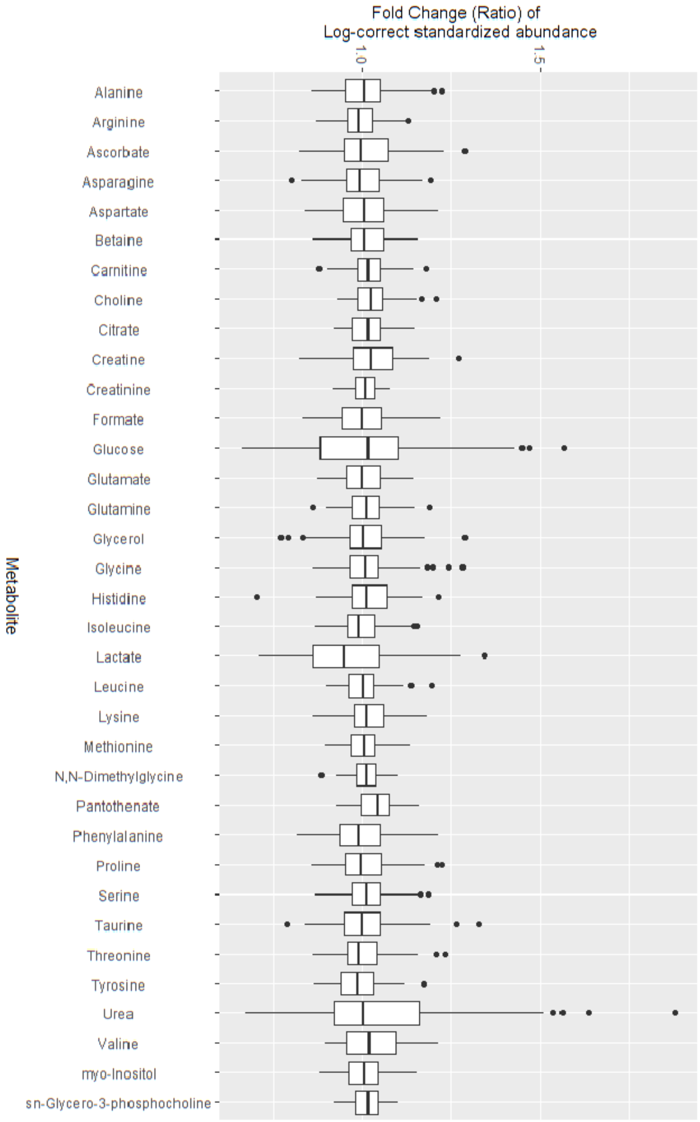

Figure S3 – Box plot representation of the studied metabolites' fold changes (concentration after NACT/concentration before NACT).

Table S1 – Average relative abundances of the serum metabolites detected by NMR before and after Neoadjuvant Chemotherapy, and fold-change (concentration after NACT/concentration before NACT).

	Before NACT		After NACT		Fold change after/before		Disease-free Survival *p*-values	
**Metabolites**	**Mean**	**SD**	**Mean**	**SD**	**Mean**	**SD**	**Prior to VIF**	**After VIF**
Alanine	0.043	0.009	0.043	0.009	-0.034	0.339	0.96	0.27
Arginine	0.008	0.002	0.008	0.002	0.074	0.445	0.31	0.73
Ascorbate	0.004	0.002	0.003	0.002	-0.046	0.779	0.25	0.62
Asparagine	0.004	0.001	0.004	0.001	0.057	0.606	0.34	0.95
Aspartate	0.005	0.002	0.005	0.002	-0.020	0.640	0.15	0.30
Betaine	0.004	0.001	0.004	0.002	-0.074	0.539	0.03	-
Carnitine	0.004	0.001	0.004	0.001	-0.171	0.420	0.17	-
Choline	0.003	0.001	0.002	0.001	-0.208	0.466	0.84	0.22
Citrate	0.007	0.002	0.006	0.002	-0.085	0.365	0.54	0.49
Creatine	0.004	0.002	0.004	0.002	-0.175	0.659	0.31	0.20
Creatinine	0.004	0.001	0.004	0.001	-0.033	0.293	0.21	0.14
Formate	0.005	0.002	0.005	0.001	0.021	0.629	0.48	-
Glucose	0.512	0.064	0.512	0.076	-0.006	0.175	0.02	-
Glutamate	0.010	0.003	0.010	0.003	0.001	0.416	0.80	0.48
Glutamine	0.044	0.008	0.043	0.010	-0.050	0.277	0.52	0.41
Glycerol	0.013	0.005	0.013	0.006	0.003	0.580	0.16	0.19
Glycine	0.033	0.012	0.032	0.013	-0.059	0.398	0.31	-
Histidine	0.006	0.002	0.006	0.002	-0.081	0.614	0.009	0.04
Isoleucine	0.007	0.002	0.007	0.002	0.041	0.475	0.01	-
Lactate	0.093	0.025	0.105	0.026	0.176	0.465	0.15	0.07
Leucine	0.007	0.002	0.007	0.002	0.011	0.398	0.008	-
Lysine	0.008	0.002	0.007	0.002	-0.112	0.407	0.48	-
Methionine	0.003	0.001	0.003	0.001	-0.004	0.435	0.91	-
N,N-Dimethylglycine	0.000	0.000	0.000	0.000	-0.096	0.462	0.88	0.23
Pantothenate	0.002	0.001	0.002	0.000	-0.331	0.482	0.31	0.77
Phenylalanine	0.002	0.001	0.002	0.001	0.089	0.858	0.22	0.94
Proline	0.027	0.009	0.026	0.009	-0.013	0.408	0.08	0.39
Serine	0.011	0.003	0.011	0.003	-0.050	0.411	0.03	0.01
Taurine	0.011	0.004	0.011	0.004	-0.013	0.572	0.13	0.03
Threonine	0.010	0.003	0.010	0.003	0.011	0.463	0.27	-
Tyrosine	0.005	0.002	0.005	0.002	0.122	0.520	0.28	-
Urea	0.055	0.029	0.051	0.026	-0.113	0.982	0.11	-
Valine	0.024	0.006	0.023	0.005	-0.107	0.411	0.47	-
myo-Inositol	0.004	0.001	0.004	0.001	-0.036	0.459	0.82	0.71
sn-Glycero-3-phosphocholine	0.005	0.001	0.005	0.001	-0.092	0.304	0.28	0.25

NACT: Neoadjuvant Chemotherapy. SD: Standard Deviation. VIF: Variance inflation factor.
